# Comparison of ambulatory blood pressure monitoring among patients with postural orthostatic tachycardia syndrome, autonomic dysfunction, and controls

**DOI:** 10.1038/s41371-025-01031-7

**Published:** 2025-05-30

**Authors:** Megan Bach, Joseph Kassab, Ahmed Mohamed Hassan, Nandan Kodur, Luke J. Laffin

**Affiliations:** 1 Department of Internal Medicine, Cleveland Clinic, Cleveland, OH USA; 2 Department of Internal Medicine, UT Southwestern Medical Center, Dallas, TX USA; 3 Lerner Research Institute Cancer Biology, Cleveland Clinic, Cleveland, OH USA; 4 Cleveland Clinic Lerner College of Medicine, Cleveland Clinic, Cleveland, OH USA; 5 Department of Cardiovascular Medicine, Cleveland Clinic, Cleveland, OH USA

**Keywords:** Diagnosis, Hypertension

## Abstract

The utility of ambulatory blood pressure monitoring (ABPM) among patients with various forms of autonomic dysfunction (AD) is unknown. Twenty-four-hour ABPM among patients with postural orthostatic tachycardia syndrome (POTS), AD without POTS, and control patients without AD were compared. Patients with AD without POTS had high rates of uncontrolled blood pressure (76%), whereas 19% of patients with POTS had uncontrolled blood pressure, suggesting ABPM may provide less value among patients with POTS.

Autonomic dysfunction (AD) refers to a range of conditions that impair the regulation of the autonomic nervous system, leading to clinical manifestations such as orthostatic hypotension, tachycardia, and gastrointestinal motility disorders. AD can arise from primary disorders of the autonomic nervous system or as a secondary response to systemic diseases [1]. Postural orthostatic tachycardia syndrome (POTS) is a form of AD and characterized by an excessive increase in heart rate upon standing, often accompanied by symptoms such as dizziness, fatigue, and lightheadedness [2]. POTS results from a failure to adequately maintain vascular tone, causing blood pooling in the lower extremities and may contribute to blood pressure (BP) fluctuations.

The American Autonomic Society and European Federation of Autonomic Societies recommend ambulatory blood pressure monitoring (ABPM) to evaluate nocturnal hypertension in patients with neurogenic orthostatic hypotension, particularly those suspected of having neurogenic supine hypertension and those undergoing treatment with anti-hypotensive agents [3]. However, there are currently no established guidelines advocating for, or against, the use of ABPM in patients diagnosed with POTS. The clinical role and potential utility of ABPM in this population remain undefined and warrants further investigation.

We sought to determine if rates of uncontrolled BP among patients undergoing 24-h ABPM were different in patients with POTS, AD without POTS, and controls (no AD.). We hypothesized that ABPM parameters would be similar between patients without AD and patients with POTS, but dissimilar among patients with AD without POTS. This was a Cleveland Clinic IRB approved, single-center, retrospective analysis of ABPM data obtained from patients at the Cleveland Clinic between January 2019 and July 2023. All ABPM studies were performed using the Oscar 2 ABPM device (SunTech Medical) and machines were programmed to measure brachial BP every 15 min during the day and every 30 min at night. Comprehensive ABPM data, as well as demographic and clinical characteristics, were collected from the electronic medical record. Determination of POTS and AD was based on a documented clinical diagnosis in the medical chart. Uncontrolled hypertension was defined as >130/80 mm Hg over 24 h, >135/85 mm Hg for the daytime average, and >120/70 mm Hg for the nighttime average. Dipping status was determined by the reduction in nighttime systolic blood pressure as a percentage of daytime blood pressure, with abnormal dipping status defined as <10% or ≥20% change. Comparisons of BP changes between cohorts were performed using one-way ANOVA while dipping status and controlled BP were analyzed using chi-square analysis. For multiple pairwise comparisons, Bonferroni correction was applied. Post-hoc pairwise comparisons were conducted using Tukey Honestly Significant Difference (HSD) test.

A total of 243 patients had ABPM performed and 62 of those patients (25.5%) had a diagnosis of AD, of which 37 (15.2%) had a specific diagnosis of POTS. Patients with POTS were predominantly female, younger, and of white race. Eighty-one percent of patients with POTS and 69% of patients without AD had controlled BP over the 24-h period, in contrast to just 24% of those with AD but without POTS (p < 0.001). Pairwise chi-square tests were performed with Bonferroni correction applied to adjust for multiple comparisons (α = 0.017). Patients with AD but without POTS had significantly higher rates of uncontrolled BP over the 24-h period compared to patients with POTS (p < 0.001) and patients without AD (p < 0.001). There was no statistically significant difference in BP control between patients with POTS and patients without AD (p = 0.460). Multivariable linear regression analysis revealed that after adjusting for sex and age, mean systolic blood pressure was lowest among patients with POTS (117.7 +/− 14.3 mm Hg) and patients without AD (131.3 +/− 13.4 mm Hg), in contrast to the 137.1 mmHg observed in those with AD without POTS (β = 3.2, p = 0.024). No significant differences were observed based on sex (β = −0.7, p = 0.698), and the age of patients was positively associated with blood pressure (β = 0.2, p = 0.004). The Tukey HSD test revealed patients with POTS had significantly lower mean systolic blood pressure than patients with AD without POTS (p < 0.001) and patients without AD (p < 0.001). There was no significant difference between patients with AD without POTS or patients without AD (p = 0.125). Abnormal dipping status was prevalent in 51% of patients with POTS, 68% of the AD without POTS cohort, and 56% of the without AD cohort. Reverse nocturnal dipping was prevalent in none of the POTS cohort, 16% of the AD without POTS cohort, and 10% of control patients. Additional data are seen in Table 1.

**Table 1:** Ambulatory Blood Pressure Monitoring Patient Characteristics and Results.

	Patients with POTS (N = 37)	Patients with autonomic dysfunction without POTS	Patients without autonomic dysfunction	P value
		(N = 25)	(N = 181)	
Age – mean (SD)	34.8 (12.4)	56.6 (16.5)	61.5 (16.5)	<0.001
Female sex – n (%)	32 (86.5)	14 (56)	103 (56.9)	0.003
BMI – mean (SD)	26.2 (7.9)	28.3 (7.3)	28.7 (7.2)	0.170
White ethnicity – n (%)	37 (100)	24 (96)	149 (82.3)	0.006
Mean % valid samples per ABPM (SD)	85 (16.9)	84 (17.6)	84 (17.1)	0.966
Patients taking antihypertensive medications – n (%)	26 (70.3)	16 (64.0)	149 (82.3)	0.045
24 h SBP in mm Hg – mean (SD)	117.7 (14.3)	137.1 (16.5)	131.3 (13.4)	<0.001
24 h DBP in mm Hg – mean (SD)	68.4 (8.1)	75.5 (10.7)	72.4 (9.3)	0.010
Patients with 24-h controlled BP – n (%)	30 (81)	6 (24)	125 (69)	<0.001
Patients with 24-h uncontrolled BP – n (%)	7 (19)	19 (76)	56 (31)	<0.001
24 h HR in bpm – mean (SD)	76.5 (7.4)	70.9 (9.6)	70.6 (10.2)	0.004
Abnormal dipping status – n (%)	19 (51)	17 (68)	99 (56)	0.413
			[n = 177]	
Non-dipping status – n (%)	17 (46)	9 (36)	66 (37)	0.206
			[n = 177]	
Reverse-dipping status – n (%)	0 (0)	4 (16)	18 (10)	0.206
			[n = 177]	
Extreme-dipping status – n (%)	2 (5)	4 (16)	15 (8)	0.206
			[n = 177]	

*ABPM* ambulatory blood pressure monitoring, *AD* autonomic dysfunction, *BMI* body mass index, *BP* blood pressures, *BPM* beats per minute, *DBP* diastolic blood pressure, *HR* heart rate, *POTS* postural orthostatic tachycardia syndrome, *SBP* systolic blood pressure, *SD* standard deviation.

Ambulatory BP monitoring reveals contrast among the patient groups. Namely, patients with POTS had significantly higher rates of controlled BP over a 24-h period, whereas the majority of patients with AD, but without POTS, had uncontrolled BP. The findings suggest that patients with AD, without POTS, may have greater degrees of impairment in BP regulation, reflecting dysautonomia and reinforcing guidelines proposing 24-h ABPM as a useful test for this patient group. The utility of 24-h ABPM among patients with POTS is less clear. Although the present data suggest a low percentage of patients with POTS have elevated BP over the 24-h period, and there was no supine hypertension or reverse nocturnal dipping among this patient group, ABPM may still offer clinical value in select cases or in the presence of coexisting conditions. No difference in non-dipping status was found between POTS patients compared to patients with AD without POTS, or patients without AD despite these individuals being younger and having lower mean systolic blood pressures. Study limitations include the inability to account for why the ABPM was ordered and the low number of patients with AD, but without POTS.

Overall, our analysis demonstrates that patients with non-POTS AD demonstrate higher rates of uncontrolled hypertension during 24-h ABPM compared with patients with POTS. Patients with POTS demonstrate BP patterns that are more similar to patients without AD. These findings corroborate guidelines recommending ABPM in patients with AD, but suggest there may be less clinical utility of ABPM in POTS patients compared with other forms of AD.
